# Intrauterine Transfusion for Rhesus Alloimmunization: A Historical Retrospective Cohort from A Single Reference Center in Brazil

**DOI:** 10.3390/jcm13051362

**Published:** 2024-02-28

**Authors:** David Baptista da Silva Pares, Gilda Helena Arruda Sousa Pacheco, Guilherme Antonio Rago Lobo, Edward Araujo Júnior

**Affiliations:** Department of Obstetrics, Paulista School of Medicine, Federal University of São Paulo (EPM-UNIFESP), São Paulo 04023-062, SP, Brazil; davidpares@uol.com.br (D.B.d.S.P.); ultragil@terra.com.br (G.H.A.S.P.); guirlobo@gmail.com (G.A.R.L.)

**Keywords:** Rh alloimmunization, intrauterine transfusion, bradycardia, umbilical cord bleeding, hydrops, fetal death

## Abstract

**Objective:** This study aimed to describe the historical experience of a single reference center in Brazil with intrauterine transfusion (IUT) for Rhesus (Rh) alloimmunization, evaluating the major complications and the perinatal outcomes of this procedure. **Methods:** This retrospective cohort study evaluated data from medical records of pregnant women between 20 and 34 weeks of gestation whose fetuses underwent IUT by cordocentesis between January 1991 and June 2021. The same experienced examiner performed all procedures. Univariate and multivariate logistic regression was used to assess the effect of fetal hydrops, duration of IUT, post-transfusion cord bleeding time, and bradycardia on death (fetal or neonatal). **Results:** We analyzed data from 388 IUTs in 169 fetuses of alloimmunized pregnant women with a mean age of 29.3 ± 5.1 years. Death and fetal hydrops were significantly associated at first IUT (*p* < 0.001). We had two cases of emergency cesarean section (mean of 0.51% per IUT) and three cases of premature rupture of the ovular membranes (mean of 0.77% per procedure). Thirty-six deaths were recorded, including 14 intrauterine and 22 neonatal. A higher percentage of neonatal deaths was observed in the group with post-transfusion cord bleeding time > 120 s (45.8%). The odds of neonatal death were 17.6 and 12.9 times higher in cases with hydrops and bradycardia than in cases without hydrops and bradycardia, respectively. The odds of death (fetal and neonatal) were 79.9 and 92.3 times higher in cases with hydrops and bradycardia than in cases without hydrops and bradycardia, respectively. **Conclusions:** The most common complications of IUT for Rh alloimmunization were post-transfusion cord bleeding, fetal bradycardia, premature rupture of ovular membranes, and emergency cesarean section. The IUT complication most associated with death (fetal and neonatal) was bradycardia, and the perinatal outcomes were worse in fetuses with hydrops.

## 1. Introduction

Perinatal hemolytic disease (PHD) or fetal erythroblastosis is an immunological disease resulting from maternal–fetal blood incompatibility. Due to a complete lack of knowledge about its etiopathogenesis, it was responsible for a high number of perinatal deaths for a long time [1]. Despite significant advances in medicine over the last five decades, this disease continues to have an ominous impact on the well-being of newborns. Notably, despite the overall reduction in incidence, there is no trend towards its eradication, mainly due to negligence in the use of adequate prophylaxis [2].

Diagnosis of Rhesus (Rh) factor alloimmunization is made by the indirect Coombs test, which is characterized by testing maternal blood for anti-erythrocyte antibodies of any origin and is not specific for anti-Rh antibodies. The antibody must be identified and titrated if positive. The major culprit in PHD is the type D antibody, and the disease is present when the dilution titer is ≥1/16 [3]. PHD is a particularly insidious hemolytic disease of the fetus and newborn, characterized by excessively rapid destruction of red blood cells, resulting in severe anemia, hyperbilirubinemia, and severe generalized edema. In its most severe form, it is caused by specific antibodies produced by the mother that enter the fetal circulation during pregnancy [4].

The concept of intrauterine transfusion (IUT) was described by Liley, who accidentally punctured the abdomen of a fetus during amniocentesis, and yellowish fluid came out. After noticing the accidental puncture, he injected radiopaque contrast and confirmed the position of the needle by radiological examination [5]. In 1983, Daffos et al. [6] described the first experience with ultrasound-guided collection of umbilical blood using a single needle. The technique was called cordocentesis and had the advantages of not contaminating fetal blood, reducing fetal loss rates, and being relatively easy to perform compared to fetoscopy. In addition to these advantages, it could be used until the end of pregnancy. Thus, it became the method of choice for obtaining fetal blood.

IUT is undoubtedly the treatment of choice for the severe form of PHD, preventing intrauterine death with survival rates of 60–90% [7,8,9]. However, this procedure is not without risk. IUT promotes the passage of fetal blood into the maternal circulation in 66% of cases, exacerbating the mother’s immune response. In addition, complications occur in approximately 2% of cases, the most common being bradycardia, hypercapnia, premature rupture of ovular membranes, and umbilical cord bleeding, sometimes resulting in fetal death [10,11]. IUT is the procedure of choice for the treatment of severe cases of Rh alloimmunization; however, it requires a curve of experience and is not without complications. Therefore, a historical cohort makes it possible to evaluate technical improvements as well as improvements in perinatal outcomes.

This study aimed to delineate the historical experience of a single reference center in Brazil with IUT for Rh alloimmunization, evaluating the major complications and the perinatal outcomes of this procedure.

## 2. Methods

### 2.1. Study Design

This retrospective cohort study aimed to evaluate data from medical records of pregnant women between 20 and 34 weeks of gestation whose fetuses underwent IUT by cordocentesis between January 1991 and June 2021, in accordance with the care criteria of the Alloimmunized Pregnant Women Care Sector of the Fetal Medicine Discipline, Department of Obstetrics, Federal University of São Paulo (UNIFESP). This study was approved by the Ethics Committee of UNIFESP (CAAE: 61118822.1.0000.5505).

### 2.2. Variables

Data collected from medical records included date of procedure, maternal age (years), number of pregnancies and parity, ethnicity (white, black, or mixed), gestational age at the time of IUT (in weeks dated by ultrasound examination), gestational age at delivery, number of days of newborn admission, number of IUTs, the volume of blood infused and withdrawn, post-transfusion cord bleeding time, indirect Coombs test score, maternal blood group, pre- and post-transfusion fetal hemoglobin levels, placenta location, and presence of fetal hydrops.

### 2.3. Technique 

All the cordocenteses were performed by the same experienced examiner, according to the following technique: location of the umbilical cord by ultrasound, preferably at its placenta insertion; identification of the three vessels, opting for the abdominal insertion and, as a last choice, the free loop of the umbilical cord, as in the case of placentas with posterior insertion. Next, using the “freehand” technique, a 20G needle was inserted until the tip was visible, preferably in the vein. At this point, if necessary, vecuronium bromide was infused, as described by Leveque et al. [12]. Before collecting 1.0 mL of fetal blood, approximately 0.5 mL of saline was infused, just enough to obtain an echogenic turbulence image to ensure correct positioning. For post-procedure hemoglobin measurement, 2–3 mL was collected, and this sample was sent to the laboratory for determination of fetal hemoglobin using an Abbott Cell-Dyn 3500 Hematology Analyzer (Ramsey, MN, USA). 

Fetal IUT was performed when hemoglobin levels were ≤10 g/dL, in accordance with Bowman [13]. Blood was then infused using a three-way stopcock system attached to the blood bag on one side, an extender attached to the puncture needle on the other, and a 20 mL syringe in the middle, which was continuously filled until the volume previously calculated for infusion was reached. In this system, the blood injection rate was approximately 5–10 mL per min due to the maximum pressure on the syringe plunger as opposed to the pressure resulting from the caliber of the extension system. Throughout the procedure, the maintenance of the vortex motion within the punctured vessel, represented by the echogenic image, and the fetal heart rate (FHR) were continuously monitored through the ultrasound. The infusion was stopped immediately if there was a change in either of these parameters. After removal of the needle, we checked for bleeding at the puncture site. If present, we measured its duration in seconds. Next, the FHR was assessed, noting the presence and duration of bradycardia, which was considered positive if the rate was <120 bpm.

From 1998 onwards, the formula described by Giannina et al. [14] was used to calculate the volume to be infused into the fetus. Between 1992 and 1997, an attempt was made to achieve half the fetal–placental volume for gestational age, as long as there was no fetal bradycardia, according to Rodeck et al. [15]. The blood used was prepared according to the following criteria: (1) fresh blood, i.e., stored for a maximum of 48 h to reduce the risk of hypercapnia; (2) O Rh negative; (3) concentrated to a hematocrit between 65% and 75% to minimize infusion; and (4) irradiated or “washed” to destroy leukocytes and reduce the incidence of fetal graft–host reaction. The hematocrit rate was calculated as a drop per day, and an increase in the peak systolic velocity of the middle cerebral artery (after the year 2000 [16]) was also taken into account for the indication of new IUT procedures.

### 2.4. Statistical Analysis

Data were first analyzed descriptively. Absolute and relative frequencies were presented for categorical variables and summary measures (mean, quartiles, minimum, maximum, and standard deviation) for numerical variables. Associations between two categorical variables were tested using the chi-squared test or, in the case of small samples, Fisher’s exact test. Comparison of means between two and more than two groups was performed using the Mann–Whitney and Kruskal–Wallis nonparametric tests, respectively. When differences in means were found after the Kruskal–Wallis test, Dunn–Bonferroni multiple comparisons were performed to maintain the overall level of significance.

Univariate and multivariate logistic regression was used to assess the effect of fetal hydrops, duration of IUT, post-transfusion cord bleeding time, and bradycardia on death (fetal or neonatal). The goodness of fit of the multivariate model was assessed using the Hosmer and Lemeshow test. In the multivariate models, predictor variables were those that were significant at 20% in the univariate analysis due to the low occurrence of deaths. Variables that were not significant at 5% were then excluded in order of significance (backward method). In addition, the goodness of fit of the final model was assessed using the Hosmer and Lemeshow test. A 5% significance level was used for all statistical tests. Analyses were performed using the SPSS 20.0.2 statistical package (IBM Corp., Armonk, NY, USA) and STATA 175 (Stata Corp., College Station, TX, USA).

## 3. Results

We analyzed data from 457 IUTs; however, we excluded 69 (15.0%) procedures with incomplete data. Therefore, we analyzed data from 388 IUTs in 169 fetuses of alloimmunized pregnant women, with a mean age of 29.3 ± 5.1 years (minimum age of 17 years and maximum age of 44 years). The number of pregnancies varied from 1 to 11; 66.3% of the study participants were multigravidas (2–4 pregnancies), and 51.5% had two IUTs. Only two patients were primigravidas, both sensitized by incompatible blood transfusions. More than half of the IUTs (56.4%) were performed between 28 and 32 weeks of gestation, with a median procedure and post-transfusion cord bleeding time of 13 min and 60 s, respectively. At the first IUT, 35.5% of fetuses had hydrops, and 50% of these fetuses died. Only 4.1% of the fetuses in the 388 IUTs had bradycardia (FHR < 120 bpm). Regarding the location of the placenta, 60.6% and 39.4% of the IUTs were performed in pregnant women with an anterior and posterior placenta, respectively (Table 1).

A significant association was observed between fetal hydrops and mortality at the first IUT (*p* < 0.001), with a higher mortality rate in cases presenting with fetal hydrops at the first IUT (50.0% vs. 5.5%). Of the deaths, 14 were intrauterine as a direct result of the procedure, and 22 were due to neonatal complications such as prematurity, severe anemia, and post-exsanguinous transfusion infection.

The placenta location was anterior in 235 cases and posterior in 153 cases. In 59% of the IUTs performed at the placenta insertion of the umbilical cord, when the placenta was posterior, fetal paralysis with the administration of curare was necessary. In 64% of the free loop punctures, this paralysis was extremely important for the IUT. 

In our series, we had two cases of emergency cesarean section, a mean of 0.51% per IUT. In one case, the ultrasound image of the puncture site showed a hyperechogenic area accompanied by intense bradycardia, which we confirmed as a hematoma at the cordocentesis site. In the second case, the fetus was severely anemic due to probable severe cord hemorrhage at the puncture site, and both fetuses died. We observed three cases of premature rupture of ovular membranes, a mean of 0.77% per procedure, occurring within 72 h of the IUT, which we classified as inherent to the procedure. Of the 36 deaths, 14 were intrauterine and 22 neonatal. 

In 51 (13.1%) IUTs, we observed post-transfusion cord bleeding at the cord puncture site of >120 s, and it was lower in IUTs where the placenta was anterior so that the cord was punctured at its insertion through the placenta. In these cases, there was no bleeding in 69% of cases, <120 s in 25% of cases, and >120 s in 25% of cases. Bradycardia remained for ≥2 min in 16 (4.1%) of the 388 IUTs. The mean procedure times for anterior placenta, posterior placenta, posterior placenta with free loop, and posterior placenta with free loop and curare were 12, 17.6, 17.1, and 18.2 min, respectively. A significant association was observed between placenta location and procedure time (*p* < 0.001), post-transfusion cord bleeding time (*p* < 0.001), post-transfusion cord bleeding time classes (*p* < 0.001), and bradycardia (*p* = 0.027) (Table 2).

In cases of fetal death, we analyzed the possible factors most associated with this outcome, as shown in Table 3. Fetal death was significantly associated with procedure times (*p* = 0.001), post-transfusion cord bleeding time (*p* = 0.001), post-transfusion cord bleeding time classes (*p* = 0.002), bradycardia (*p* < 0.001), and hydrops (*p* < 0.001). Therefore, procedure and post-transfusion cord bleeding times were longer in the fetal death groups. The percentages of fetal deaths were also lower in the groups with no bleeding (3.8%), no bradycardia (6.4% vs. 71.4%), and no hydrops (0.8% vs. 52.0%). Contrastingly, the percentage of fetal deaths was higher in the group with a post-transfusion cord bleeding time > 120 s (31.6%).

Univariate and multivariate logistic regression models were then fitted with fetal death as the dependent variable. In the logistic regression model, the exponentiated coefficients were interpreted as odds ratios. In this study, the odds consisted of the ratio of the probability of fetal death to no fetal death. For post-transfusion cord bleeding time, the numerical form was considered in the initial multivariate model because it was more statistically significant in the univariate model than in the categorical form. These data are shown in Table 4. Hydrops, procedure and post-transfusion cord bleeding times (in numerical form), and bradycardia (significant at 10% in the univariate models) were considered in the initial multivariate model. Only hydrops remained significant in the final model (*p* < 0.001). Thus, the chance of fetal death in cases with hydrops was 131.1 times higher than in cases without this condition.

Table 5 shows the associations between neonatal death and procedure time (*p* = 0.004), post-transfusion cord bleeding time (*p* < 0.001), post-transfusion cord bleeding time classes (*p* < 0.001), bradycardia (*p* < 0.001), hydrops (*p* < 0.001), and placenta location (*p* = 0.004). Therefore, procedure and post-transfusion cord bleeding times were longer in the neonatal death groups. The percentages of neonatal deaths were also lower in the groups with no bleeding (7.2%), no bradycardia (9.7% vs. 80.0%), no hydrops (4.7% vs. 57.1%), and anteriorly located placenta (9.1% vs. at least 33.3%). In contrast, a higher percentage of neonatal deaths was observed in the group with post-transfusion cord bleeding time > 120 s (45.8%).

According to Table 6, hydrops, procedure and post-transfusion cord bleeding times (in numerical form), bradycardia, and placenta location were included in the initial multivariate model (significant at 10% in the univariate models). Hydrops (*p* < 0.001) and bradycardia (*p* = 0.010) remained significant in the final model. Thus, the odds of neonatal death were 17.6 times higher in cases with hydrops than in cases without hydrops. Similarly, the odds of neonatal death were 12.9 times higher in cases with bradycardia than in cases without this condition.

Table 7 shows the associations between death (fetal and neonatal) and procedure time (*p* = 0.001), post-transfusion cord bleeding time (*p* < 0.001), post-transfusion cord bleeding time classes (*p* < 0.001), bradycardia (*p* < 0.001), hydrops (*p* < 0.001), and placenta location (*p* = 0.019). Procedure and post-transfusion cord bleeding times were longer in the death groups. The percentages of deaths were also lower in the groups with no bleeding (10.5%), no bradycardia (14.3% vs. 93.3%), no hydrops (3.9% vs. 75.6%), and anteriorly located placenta (14.3%). Conversely, a higher percentage of deaths was observed in the group with post-transfusion cord bleeding time >120 s (56.7%) and posteriorly located placenta with a free loop (39.3%).

According to Table 8, hydrops, procedure and post-transfusion cord bleeding times (in numerical form), bradycardia, and placenta location were included in the initial multivariate model (significant at 20% in the univariate models). Hydrops (*p* < 0.001) and bradycardia (*p* = 0.001) remained significant in the final model. Thus, the odds of death were 79.9 times higher in cases with hydrops than in cases without hydrops. Similarly, the odds of death (fetal and neonatal) were 92.3 times higher in cases with bradycardia than in cases without bradycardia.

## 4. Discussion

Although immunoprophylaxis with the use of anti-Rh immunoglobulin has significantly reduced the emergence of new cases of maternal immunization, Rh alloimmunization still exists in developing and developed countries, and without a doubt, this is the pathology responsible for the vast majority of fetuses with moderate to severe anemia, requiring IUT therapy as the only treatment to prevent the death of the fetus [17].

In our study, we performed 388 IUTs, with an average of 2.3 procedures per fetus. In the literature, we found only one referral service in the Netherlands with the highest number of IUTs performed (1852 procedures between 1987 and 2016) over a similar period to our study [18]. 

In 96.5% of our cases, the anti-erythrocyte antibody found was anti-D, which is the most immunogenic and causes the most fetal hemolysis [13]. The mean maternal age was 29.3 ± 5.1 years; 66.3% of the pregnant women were multiparous, and 32.5% were large multiparous. Alloimmunization is undoubtedly a feature of this population, as the natural history of the disease is progressive and worsens with the number of pregnancies.

Of the 388 IUTs performed in service, 123 were performed on fetuses with hydrops. Of these, 50% had regression of hydrops and survived. This survival rate we obtained is slightly lower than that reported in the literature, which varies between 61.7% and 78% [19,20], and close to other authors who obtained survival rates between 44% and 55% [21,22]; in only one study, the survival rate was 90% [23]. We believe that in our series, the aggravating factors for these fetuses were the delay in diagnosing severe anemia before the hydropic state and the delay in referral to our center. Notably, in two cases, the same pregnant woman attended twice in our service. In both cases, at the first referral, their fetuses had severe hydrops, and they submitted to IUTs; however, the fetuses did not survive. However, both pregnant women were told to come to our service as soon as the pregnancy was diagnosed in the subsequent pregnancy. In both cases, we were able to perform the first IUT before the onset of hydrops but with severe anemia, and these two fetuses survived.

We achieved an overall fetal survival rate of 79%. This rate is relatively slightly lower than that reported in the literature, which ranges from 88% to 97% in centers with the largest caseloads [20,24]. Undoubtedly, the presence of a higher number of deaths in the fetuses with a hydropic condition lowered our overall survival rate, as 35% of our fetuses had severe hydrops, compared to 21% in the Van Kamp et al. study [20] and only 12% in the Pasman et al. study [25]. Regarding the survival rate of fetuses without hydrops at the first IUT, our percentage was 94.5%, with a statistically significant difference compared to fetuses with hydrops. This rate is similar to that observed in the literature [18,20,26], confirming the influence of fetuses with severe anemia in reducing our overall survival rate. 

In Turkey, Savkli et al. [27] evaluated the results of 110 IUTs performed on 42 fetuses between 2015 and 2018, with a fetal survival rate of 80.9% and a procedure-related complication rate of 12.7%. In 2020, Raashid et al. [28] published Pakistan’s 12-year experience of performing IUT for Rh alloimmunization. Independent of hydrops, the authors found an overall survival rate of 71.6% and a procedure-related fetal loss of 14.6%. In a study from 19 Japanese centers between 2011 and 2015, Mizuuchi et al. [29] evaluated 100 IUTs performed on 66 fetuses diagnosed with anemia not only due to Rh alloimmunization. The authors found a survival rate of 77.3% in those who did not have hydrops at the time of the first procedure and 64% in those who already had hydrops. The most common adverse outcome was post-transfusion cord bleeding, which occurred in 40% of cases, and in 25 of these, emergency cesarean section was indicated for fetal acute distress.

We used univariate analysis to identify possible associations between IUT and fetal death. We found an association with procedure time, post-transfusion cord bleeding time, bradycardia, and hydrops. Van Kamp et al. [20] also point out that free loop punctures, which are more related to the posterior location of the placenta, are associated with these complications. Death occurred in 6.4% of fetuses without bradycardia and in 71.4% with bradycardia. This association was also positive when the post-transfusion cord bleeding time was >120 s, with a 31.6% death rate. When the post-transfusion cord bleeding time was <120 s, the fetal death rate was 10.4%, and when this time was absent, the fetal death rate was 3.8%.

Analyzing all the data together, we can conclude that the location of the placenta may be a relevant factor in complications arising from IUT because when the umbilical cord is punctured in the posterior uterine wall, it is transamniotic and therefore more often in a free loop, and it is more associated with the presence of fetal bradycardia, with a consequent greater chance of fetal death. Zwiers et al. [24], in their report of a large series of 1678 procedures, also mention free loop transamniotic puncture as a negative risk factor in the procedure, along with operator experience. In this sense, in our series, all procedures were performed by a single operator with a learning curve of three decades, but this fact was not taken into account in our study.

In assessing the risk of IUT, we considered emergency cesarean section, premature rupture of ovular membranes, and prolonged bradycardia (FHR < 120 bpm and >120 s) to be the most important. Thus, we found a complication rate per fetus of 11.2% and 5.3% per procedure, while Pasman et al. [25], Deka et al. [23], van Kamp et al. [20], Tiblad et al. [26], and Zwiers et al. [18] found rates between 6.1% and 8.8% per fetus and 1.5 and 2.97% per procedure. Our complication rates were higher than those found in the literature, which we ascribe to the great differences in the population studied; they differed mainly in the number of fetuses with severe hydrops on arrival at our service. In 35% of our fetuses, hydrops was already present at the first IUT. In Pasman et al.’s study [25], hydrops was present in only 8% of the fetuses, with the important value being the only factor in the occurrence of adverse effects assessed by multivariate analysis. In the large series of Zwiers et al. [18], 13% of the fetuses had hydrops. The closest case study to ours is also from a developing country, published by Deka et al. [23], in which 22% of the fetuses had hydrops. Our overall survival rate of 79% was undoubtedly compromised by the delay in the presentation of these pregnant women. This is demonstrated by the 94.5% survival rate for those nonhydropic but severely anemic fetuses that required correction by IUT.

In our study, we could assess which variables were most associated with these adverse outcomes and showed that posterior placenta location and transamniotic puncture might be more associated with bradycardia, which is associated with a 92-fold increased risk of fetal death. Similarly, we showed that hydrops is associated with an 80-fold increased risk of death, further emphasizing the importance of referring alloimmunized pregnant women to a tertiary center promptly.

In a recent systematic review, 60 articles were selected to assess the prenatal treatment landscape and outcomes of Rh- and K-mediated PHD. The prevalence was 0.047% and 0.006% for Rh- and K-mediated PHD, respectively. The most commonly reported antenatal treatment was IUT with a median frequency of 13.0%. The mean gestational age at first IUT ranged from 25 to 27 weeks. The rate of hydrops in pregnancies with Rh-mediated PHD treated with IUT was 14.8% (range, 0–50%) and 39.2% in K-mediated PHD [30]. In another recent systematic review, 38 studies with 2323 fetuses and 5688 IUTs from 18 countries were analyzed between January 1990 and December 2021. The mean gestational age at first IUT was 26.21 weeks, pre-transfusion hemoglobin was 6.01 g/dL, and hydrops at diagnosis was reported in 32.36% of cases. Perinatal survival was 82.39%, the mean gestational age at delivery was 33.67 weeks, and the mean hemoglobin level at birth was 11.62 g/dL. The perinatal survival rate was 82.4% [31]. A retrospective cohort study evaluated the perinatal outcomes of Rh alloimmunization in a single university hospital in China between January 2001 and December 2018. A total of 244 IUTs were performed in 81 fetuses from 80 pregnancies. Anti-RhD was the main etiology of PHD requiring IUT (71.6%). Fetal survival was 90.1%. The survival rate of hydropic fetuses was significantly lower than that of nonhydropic fetuses (61.2% vs. 95.6%). Neonatal survival was 98.6% [32].

Our study had limitations. It is well known that IUT requires a learning curve, and it is expected that better perinatal outcomes will occur over time. However, this study does not allow us to answer this question. Regarding the earliest selection of Rh-alloimmunized pregnant women for IUT, it is now known that the best method is PVS-MCA; however, this method was described in 2000 and our cohort began in 1992.

## 5. Conclusions

In summary, in our historical cohort (January 1991 and June 2021), the most common complications of IUT for Rh alloimmunization were post-transfusion cord bleeding, fetal bradycardia, premature rupture of ovular membranes, and emergency cesarean section. The IUT complication most associated with death (fetal and neonatal) was bradycardia, and the perinatal outcomes were worse in fetuses with hydrops.

## Figures and Tables

**Table 1:** Maternal and intrauterine transfusion characteristics.

Maternal characteristics (*N* = 169)	
Age (years)	
Mean ± SD	29.3 ± 5.1
Median (IIQ)	29.0 (26.0–32.5)
Number of pregnancies, *N* (%)	
Primigravida	2 (1.2)
Multigravida (2 to 4 pregnancies)	112 (66.3)
Large multigravida (≥5 pregnancies)	55 (32.5)
Intrauterine transfusions, *N* (%)	
1	32 (18.9)
2	87 (51.5)
3	26 (15.4)
4	17 (10.1)
5	7 (4.1)
Fetal hydrops at the time of the first intrauterine transfusion, *N* (%)	
Yes	60 (35.5)
No	109 (64.5)
Death, *N* (%)	
Fetal hydrops at the time of the first intrauterine transfusion	30/60 (50.0)
No fetal hydrops at the time of the first intrauterine transfusion	6/109 (5.5%)
Intrauterine transfusions (*N* = 388)	
Gestational age (weeks), *N* (%)	
19–23	24 (6.2)
24–27	100 (25.8)
28–32	219 (56.4)
33–35	45 (11.6)
Procedure time (minutes)	
Mean ± SD	14.3 ± 4.8
Median (IIQ)	13.0 (10.0–17.0)
Post-transfusion cord bleeding time (seconds)	
Mean ± SD	54.9 ± 56.5
Median (IIQ)	60.0 (0.0–100.0)
Post-transfusion cord bleeding time (seconds)	
No bleeding	174 (44.8)
≤120 s	163 (42.0)
>120 s	51 (13.1)
Hydrops, *N* (%)	
No	265 (68.3)
Yes	123 (31.7)
Bradycardia, *N* (%)	
No	372 (95.9)
Yes	16 (4.1)
Location of placenta,—4 classes *N* (%)	
Anterior	235 (60.6)
Posterior	21 (5.4)
Posterior with free loop	66 (17.0)
Posterior with free loop and use of curare	66 (17.0)
Location of placenta,—2 classes *N* (%)	
Anterior	235 (60.6)
Posterior	153 (39.4)
Curarization	
Placental insertion	30/51 (58.8)
Free handle	36/101 (35.6)
Abdominal insertion	0/1 (0.0)

IIQ: interquartile range; SD: standard deviation.

**Table 2:** Mean time and complications of intrauterine transfusion in relation to placental position.

	Location of the Placenta				Total	*p*
	Anterior (*N* = 235; 60.6%)	Posterior Insertion (*N* = 21; 5.4%)	Posterior with Free Loop (*N* = 66; 17.0%)	Posterior with Free Loop and Use of Curare (*N* = 66; 17.0%)		
Procedure time/min, mean ± SD	12.0 ± 3.2	17.6 ± 3.3	17.1 ± 4.6	18.2 ± 5.7	14.3 ± 4.8	<0.001 ^c^
Post-transfusion cord bleeding time/s, mean ± SD	25.8 ± 43.8	102.6 ± 37.9	98.8 ± 46.2	99.6 ± 41.4	54.9 ± 56.5	
Post-transfusion cord bleeding time/s—classification, *N* (%)						<0.001 ^a^
No bleeding					174 (44.8)	
≤120 s					163 (42.0)	
>120 s					51 (13.1)	
Bradycardia, *N* (%)						0.027 ^b^
No	228 (97.0)	18 (85.7)	61 (92.4)	65 (98.5)	372 (95.9)	
Yes	7 (3.0)	3 (14.3)	5 (7.6)	1 (1.5)	16 (4.1)	

SD: standard deviation. Chi-square (^a^), Fisher’s Exact (^b^), and Kruskal–Wallis (^c^) tests.

**Table 3:** Mean procedure time, intrauterine transfusion complications, and placenta location in relation to fetal death.

Fetal Death				
	No (*N* = 133; 90.5%)	Yes (*N* = 14; 9.5%)	Total	*p*
Procedure time/min, mean ± SD	13.3 ± 5.1	18.2 ± 6.2	13.9 ± 5.5	0.001 ^b^
Post-transfusion cord bleeding time/s, mean ± SD	40.3 ± 52.3	100.7 ± 68.0	46.0 ± 56.0	0.001 ^b^
Post-transfusion cord bleeding time/s—classes, *N* (%)				0.002 ^a^
No bleeding	77 (96.3)	3 (3.8)	80 (100.0)	
≤120 s	43 (89.6)	5 (10.4)	48 (100.0)	
>120 s	13 (68.4)	6 (31.6)	19 (100.0)	
Bradycardia, *N* (%)				<0.001 ^a^
No	131 (93.6)	9 (6.4)	140 (100.0)	
Yes	2 (28.6)	5 (71.4)	7 (100.0)	
Hydrops, *N* (%)				<0.001 ^a^
No	212 (99.2)	1 (0.8)	122 (100.0)	
Yes	12 (48.0)	13 (52.0)	25 (100.0)	
Location of the placenta				0.103 ^a^
Anterior	90 (93.8)	6 (6.3)	96 (100.0)	
Posterior	4 (80.0)	1 (20.0)	5 (100.0)	
Posterior with free loop	16 (80.0)	4 (20.0)	20 (100.0)	
Posterior with free loop and curare use	23 (88.5)	3 (11.5)	26 (100.0)	

SD: standard deviation. Chi-square (^a^), Fisher’s Exact (^b^), and Kruskal–Wallis.

**Table 4:** Logistic regression results for fetal death.

	Univariate Model		Initial Multivariate		Multivariate Final	
	Gross OR (95% CI)	*p*	Adjusted OR (95% CI)	*p*	OR (95% CI)	*p*
Hydrops (ref. = No)	131.08 (15.75–1090.64)	<0.001	80.26 (9.08–709.25)	<0.001	131.08 (15.75–1090.64)	<0.001
Procedure time/min	1.12 (1.02–1.23)	0.013	1.01 (0.91–1.13)	0.852	-	-
Post-transfusion cord bleeding time/s	1.02 (1.01–1.03)	0.001	1.01 (0.99–1.02)	0.401	-	-
Post-transfusion cord bleeding time/s—classification (ref. = no bleeding)		0.005		-		-
≤120 s	2.98 (0.68–13.10)	0.147	-	-	-	-
>120 s	11.85 (2.63–53.38)	0.001	-	-	-	-
Bradycardia (ref. no)	36.39 (6.18–214.38)	<0.001	4.21 (0.38–46.26)	0.240	-	-
Location of the placenta (ref. = anterior)		0.249		-		-
Posterior	3.75 (0.36–39.01)	0.269	-	-	-	-
Posterior with free loop	3.75 (0.95–14.79)	0.059	-	-	-	-
Posterior with free loop curare use	1.96 (0.45–8.42)	0.367	-	-	-	-

CI: confidence interval; OR: odds ratio.

**Table 5:** Mean procedure time, intrauterine transfusion complications, and placenta location in relation to neonatal death.

Neonatal Death				
	No (*N* = 133; 85.8%)	Yes (*N* = 22; 14.2%)	Total	*p*
Procedure time/min, mean ± SD	13.3 ± 5.1	16.7 ± 5.8	13.8 ± 5.3	0.004 ^c^
Post-transfusion cord bleeding time/s, mean ± SD	40.3 ± 52.3	102.8 ± 70.3	49.0 ± 58.9	<0.001 ^c^
Post-transfusion cord bleeding time/s—classes, *N* (%)				<0.001 ^a^
No bleeding	77 (92.8)	6 (7.2)	83 (100.0)	
≤120 s	43 (89.6)	5 (10.4)	48 (100.0)	
>120 s	13 (54.2)	11 (45.8)	24 (100.0)	
Bradycardia, *N* (%)				<0.001 ^b^
No	131 (90.3)	14 (9.7)	145 (100.0)	
Yes	2 (20.0)	8 (80.0)	10 (100.0)	
Hydrops, *N* (%)				<0.001 ^b^
No	121 (95.3)	6 (4.7)	127 (100.0)	
Yes	12 (42.9)	16 (57.1)	28 (100.0)	
Location of the placenta				0.004 ^b^
Anterior	90 (90.9)	9 (9.1)	99 (100.0)	
Posterior	4 (57.1)	3 (42.9)	7 (100.0)	
Posterior with free loop	16 (66.7)	8 (33.3)	24 (100.0)	
Posterior with free loop and curare use	23 (92.0)	2 (8.0)	25 (100.0)	

SD: standard deviation. Chi-square (^a^), Fisher’s Exact (^b^), and Kruskal–Wallis (^c^) tests.

**Table 6:** Logistic regression results for neonatal death.

	Univariate Model		Initial Multivariate		Multivariate Final	
	Gross OR (95% CI)	*p*	Adjusted OR (95% CI)	*p*	Adjusted OR (95% CI)	*p*
Hydrops (ref. = No)	26.89 (8.86–81.59)	<0.001	19.46 (5.13–73.86)	<0.001	17.57 (5.41–57.12)	<0.001
Procedure time/min	1.10 (1.02–1.19)	0.020	1.02 (0.92–1.13)	0.657	-	-
Post-transfusion cord bleeding time/s	1.02 (1.01–1.03)	<0.001	1.00 (0.99–1.02)	0.701	-	-
Post-transfusion cord bleeding time/s —classes (ref. = no bleeding)		<0.001		-		
						-
≤120 s	1.49 (0.43–5.18)	0.528	-	-	-	-
>120 s	10.86 (3.42–34.48)	<0.001	-	-	-	-
Bradycardia (ref. No)	37.43 (7.23–193.84)	<0.001	5.75 (0.41–79.82)	0.193	12.94 (1.83–91.35)	0.010
Location of the placenta (ref. = anterior)		0.005		0.246		-
Posterior	7.50 (1.45–38.91)	0.016	4.19 (0.28–63.67)	0.302	-	-
Posterior with free loop	5.00 (1.68–14.88)	0.004	3.77 (0.52–27.13)	0.188	-	-
Posterior with free loop and curare use	0.87 (0.18–4.30)	0.864	0.59 (0.05–7.70)	0.690	-	-

CI: confidence interval; OR: odds ratio.

**Table 7:** Mean procedure time, intrauterine transfusion complications, and placenta location in relation to death (fetal and neonatal).

	Death		Total	*p*
	No (*N* = 133; 78.7%)	Yes (*N* = 36; 21.3%)		
Procedure time/min, mean ± SD	13.5 ± 5.3	16.7 ± 5.7	14.2 ± 5.5	0.001 ^c^
Post-transfusion cord bleeding time/s, mean ± SD	40.0 ± 51.2	103.0 ± 70.6	53.4 ± 61.4	<0.001 ^c^
Post-transfusion cord bleeding time/s—classification, *N* (%)				<0.001 ^a^
No bleeding	77 (89.5)	9 (10.5)	86 (100.0)	
≤120 s	43 (81.1)	10 (18.9)	53 (100.0)	
>120 s	13 (43.3)	17 (56.7)	30 (100.0)	
Bradycardia, *N* (%)				<0.001 ^b^
No	132 (85.7)	22 (14.3)	154 (100.0)	
Yes	1 (6.7)	14 (93.3)	15 (100.0)	
Hydrops, *N* (%)				<0.001 ^a^
No	123 (96.1)	5 (3.9)	128 (100.0)	
Yes	10 (24.4)	31 (75.6)	41 (100.0)	
Location of the placenta				0.019 ^a^
Anterior	90 (85.7)	15 (14.3)	105 (100.0)	
Posterior	5 (62.5)	3 (37.5)	8 (100.0)	
Posterior with free loop	17 (60.7)	11 (39.3)	28 (100.0)	
Posterior with free loop and curare use	21 (75.0)	7 (25.0)	28 (100.0)	

SD: standard deviation. Chi-square (^a^), Fisher’s Exact (^b^), and Kruskal–Wallis (^c^) tests.

**Table 8:** Logistic regression results for death (fetal and neonatal).

	Univariate Model		Initial Multivariate		Multivariate Final	
	Gross OR (95% CI)	*p*	Adjusted OR (95% CI)	*p*	Adjusted OR (95% CI)	*p*
Hydrops (ref. = no)	76.26 (24.30–239.28)	<0.001	208.07 (30.01–1442.63)	<0.001	79.90 (20.34–313.95)	<0.001
Procedure time/min	1.10 (1.03–1.18)	0.005	0.91 (0.81–1.03)	0.143	-	-
Post-transfusion cord bleeding time/s	1.02 (1.01–1.02)	<0.001	0.99 (0.98–1.01)	0.554	-	-
Post-transfusion cord bleeding time/s—classes (ref. = no bleeding)		<0.001		-		-
≤120 s	1.99 (0.75–5.27)	0.167	-	-	-	-
>120 s	11.19 (4.12–30.38)	<0.001	-	-	-	-
Bradycardia (ref. No)	84.00 (10.51–671.27)	<0.001	1075.77 (12.58–91,979.19)	0.002	92.27 (7.21–1180.55)	0.001
Location of the placenta (ref. = anterior)				0.106		-
Posterior	3.60 (0.78–16.66)		0.73 (0.03–20.12)	0.851	-	-
Posterior with free loop	3.88 (1.52–9.89)		16.61 (1.37–200.90)	0.027	-	-
Posterior with free loop and curare use	2.00 (0.72–5.52)		20.95 (1.42–309.77)	0.027	-	-

CI: confidence interval; OR: odds ratio.

## Data Availability

The data presented in this study are available on request from the corresponding author.

## References

[1] Jackson M.E., Baker J.M. (2021). Hemolytic Disease of the Fetus and Newborn: Historical and Current State. Clin. Lab. Med..

[2] van’t Oever R.M., Zwiers C., de Winter D., de Haas M., Oepkes D., Lopriore E., Verweij E.J.J. (2022). Identification and management of fetal anemia due to hemolytic disease. Expert Rev. Hematol..

[3] Sahoo T., Sahoo M., Gulla K.M., Gupta M. (2020). Rh Alloimmunisation: Current Updates in Antenatal and Postnatal Management. Indian J. Pediatr..

[4] Iskaros J., Kingdom J., Morrison J.J., Rodeck C. (1998). Prospective non-invasive monitoring of pregnancies complicated by red cell alloimmunization. Ultrasound. Obstet. Gynecol..

[5] Liley A. (1963). Intrauterine transfusion of the foetus in haemolytic disease. BMJ.

[6] Daffos F., Cappella-Pavlovsk M., Forestier F. (1983). A new procedure for fetal blood sampling in utero: Preliminary results of 53 cases. Am. J. Obstet. Gynecol..

[7] Plöckinger B., Strümpflen I., Deutinger J., Bernaschek G. (1994). Diagnosis and treatment of fetal anemia due to isoimmunization. Arch. Gynecol. Obstet..

[8] Ramírez-Robles L.J., Gómez-Partida G., Guevara-Rubio G., Velázquez-Gómez L. (2010). Intrauterine transfusión in alloimmunization Rh in México 1987–2008. Ginecol. Obstet. Mex..

[9] Al-Riyami A.Z., Al-Salmani M., Al-Hashami S.N., Al-Mahrooqi S., Al-Marhoobi A., Al-Hinai S., Al-Hosni S., Panchatcharam S.M., Al-Arimi Z.A. (2018). Intrauterine Fetal Blood Transfusion: Descriptive study of the first four years’ experience in Oman. Sultan. Qaboos. Univ. Med. J..

[10] Newnham J.P., Phillips J.M., Stock R. (1992). Intrauterine intravascular transfusion for fetal haemolytic anaemia: The Western Australian experience. Med. J. Aust..

[11] Alkhaibary A., Ali M., Tulbah M., Al-Nemer M., Khan R.M., Al Mugbel M., Al Sahan N., Hassounah M.M., Alshammari W., Kurdi W.I. (2021). Complications of intravascular intrauterine transfusion for Rh alloimmunization. Ann. Saudi Med..

[12] Leveque C., Murat I., Toubas F., Poissonnier M., Brossard Y., Sanint-Maurice C. (1992). Fetal Neuromuscular Blockade with Vecuronium Bromide: Studies during intravascular intrauterine Transfusion in Isoimmunized Pregnancies. Anesthesiology.

[13] Bowman J.M., Creast R.K., Resnick R., Greene M.F., Iams J.D., Lockwood C.J., Moore T.R. (1999). Hemolytic disease (Erythroblastosis Fetalis). Creasy and Resnik’s Maternal-Fetal Medicine: Principles and Practice.

[14] Giannina G., Moise K., Dorman K. (1998). A simple method to estimate volume for fetal intravascular transfusions. Fetal. Diagn. Ther..

[15] Rodeck C., Nicolaides K., Warsof S. (1984). The management of severe rhesus isoimmunization by fetoscopie intravacular transfusions. Am. J. Obstet. Gynecol..

[16] Mari G., Deter R.L., Carpenter R.L., Rahman F., Zimmerman R., Moise K.J., Dorman K.F., Ludomirsky A., Gonzalez R., Gomez R. (2000). Noninvasive diagnosis by Doppler ultrasonography of fetal anemia due to maternal red-cell alloimmunization. Collaborative Group for Doppler Assessment of the Blood Velocity in Anemic Fetuses. N. Engl. J. Med..

[17] Pegoraro V., Urbinati D., Visser G.H.A., Di Renzo G.C., Zipursky A., Stotler B.A., Spitalnik S.L. (2020). Hemolytic disease of the fetus and newborn due to Rh(D) incompatibility: A preventable disease that still produces significant morbidity and mortality in children. PLoS ONE.

[18] Zwiers C., Oepkes D., Lopriore E., Klumper F., Haas M., Van Kamp I. (2018). The near disappearance of fetal hydrops in relation to current state-of-the-art management of red cell alloimmunization. Prenat. Diagn..

[19] Papantoniou N., Stavros S., Antsaklis A. (2013). Therapeutic management of fetal anemia: Review of standard practice and alternative treatment options. J. Perinat. Med..

[20] Van Kamp I., Klumper F., Oepkes D., Meerman R., Scherjon S., Vandenbussche F., Kanhai H.H. (2005). Complications of intrauterine intravascular transfusion for fetal anemia due to maternal re-cell alloimmunization. Am. J. Obstet. Gynecol..

[21] Cabral A., Taveira M., Lopes A., Pereira K., Leite H. (2001). Intrauterine Transfusion in Maternal Rh Immunization. RBGO.

[22] Sánchez-Durán M., Higueras M., Halajdian-Madrid C., Garcia M., Bernabeu-García A., Maiz N., Nogués N., Carreras E. (2019). Management and outcome of pregnancies in women with red cell isoimunization: A 15-year observational study from a tertiary care university hospital. BMC Pregnancy Childbirth.

[23] Deka D., Dadhwal V., Sharma A., Shende U., Agarwal S., Agarwal R., Vanamail P. (2015). Perinatal survival and procedure-related complications after intrauterine transfusion for red cell alloimunization. Arch. Gynecol. Obstet..

[24] Zwiers C., Lindenburg I., Klumper F., de Haas M., Oepkes D., van Kamp I. (2017). Complications of intrauterine intravascular blood transfusion: Lessons learned after 1678 procedures. Ultrasound. Obstet. Gynecol..

[25] Pasman S., Claes L., Levi L., Van Schoubroeck D., Debeer A., Emonds M., Geuten E., De Catte L., Devlieger R. (2015). Intrauterine transfusion for fetal anemia due to red blood cell alloimmunization: 14 years experience in Leuven. Fcts. Views Obgyn..

[26] Tiblad E., Kublickas M., Ajne G., Bui T.H., Ek S., Karlsson A., Wikman A., Westgren M. (2011). Procedure-related complications and perinatal outcome after intrauterine transfusions in red cell alloimmunization in Stockholm. Fetal. Diagn. Ther..

[27] Savkli A., Cetin B., Acar Z., Ozcose Z., Behram M., Çaypinar S.S., Tayyar A., Yüksel M.A. (2020). Perinatal outcomes of intrauterine transfusion for foetal anaemia due to red blood cell alloimmunization. J. Obstet. Gynaecol..

[28] Raashid Y., Ayesha A., Ehsan Y., Jafri H., Waheed I., Mason G., Majeed N. (2020). Intrauterine fetal blood transfusion (IUBT) for Rh incompatibility—12 years’ experience from Pakistan. J. Coll. Physicians Pk..

[29] Mizuuchi M., Murotsuki J., Ishii K., Yamamoto R., Sasahara J., Wada S., Takahashi Y., Nakata M., Murakoshi T., Sago H. (2021). Nationwide survey of intrauterine blood transfusion for fetal anemia in Japan. J. Obstet. Gynecol. Res..

[30] de Winter D.P., Kaminski A., Tjoa M.L., Oepkes D. (2023). Hemolytic disease of the fetus and newborn: Systematic literature review of the antenatal landscape. BMC Pregnancy Childbirth.

[31] Donepudi R.V., Javinani A., Mostafaei S., Habich A., Miller J., Chmait R.H., Shamshirsaz A.A. (2023). Perinatal survival following intrauterine transfusion for red cell alloimmunized pregnancies: Systematic review and meta-regression. Am. J. Obstet. Gynecol..

[32] Pan W., Wu H., Chen J., Mo X., Wang H., Fang Q., Li Y., Huang Y. (2023). Fetal and neonatal outcome in severe alloimmunization managed with intrauterine transfusion: 18-year experience in a tertiary referral hospital in China. Front. Pediatr..

